# Vertebrates on the brink as indicators of biological annihilation and the sixth mass extinction

**DOI:** 10.1073/pnas.1922686117

**Published:** 2020-06-01

**Authors:** Gerardo Ceballos, Paul R. Ehrlich, Peter H. Raven

**Affiliations:** ^a^Instituto de Ecología, Universidad Nacional Autónoma de México, 04510 Ciudad de México, México;; ^b^Center for Conservation Biology, Department of Biology, Stanford University, Stanford, CA 94304;; ^c^Plant Science Department, Missouri Botanical Garden, St. Louis, MO 63110

**Keywords:** endangered species, sixth mass extinction, population extinctions, conservation, ecosystem services

## Abstract

The ongoing sixth mass species extinction is the result of the destruction of component populations leading to eventual extirpation of entire species. Populations and species extinctions have severe implications for society through the degradation of ecosystem services. Here we assess the extinction crisis from a different perspective. We examine 29,400 species of terrestrial vertebrates, and determine which are on the brink of extinction because they have fewer than 1,000 individuals. There are 515 species on the brink (1.7% of the evaluated vertebrates). Around 94% of the populations of 77 mammal and bird species on the brink have been lost in the last century. Assuming all species on the brink have similar trends, more than 237,000 populations of those species have vanished since 1900. We conclude the human-caused sixth mass extinction is likely accelerating for several reasons. First, many of the species that have been driven to the brink will likely become extinct soon. Second, the distribution of those species highly coincides with hundreds of other endangered species, surviving in regions with high human impacts, suggesting ongoing regional biodiversity collapses. Third, close ecological interactions of species on the brink tend to move other species toward annihilation when they disappear—extinction breeds extinctions. Finally, human pressures on the biosphere are growing rapidly, and a recent example is the current coronavirus disease 2019 (Covid-19) pandemic, linked to wildlife trade. Our results reemphasize the extreme urgency of taking much-expanded worldwide actions to save wild species and humanity’s crucial life-support systems from this existential threat.

## Significance

The ongoing sixth mass extinction may be the most serious environmental threat to the persistence of civilization, because it is irreversible. Thousands of populations of critically endangered vertebrate animal species have been lost in a century, indicating that the sixth mass extinction is human caused and accelerating. The acceleration of the extinction crisis is certain because of the still fast growth in human numbers and consumption rates. In addition, species are links in ecosystems, and, as they fall out, the species they interact with are likely to go also. In the regions where disappearing species are concentrated, regional biodiversity collapses are likely occurring. Our results reemphasize the extreme urgency of taking massive global actions to save humanity’s crucial life-support systems.

During the more than 4.5 billion years of Earth’s history, there has never been a richness of life comparable to that which exists today (1). Although there have been five mass extinction episodes during the last 450 million years, each destroying 70 to 95% of the species of plants, animals, and microorganisms that existed earlier (2–4), life has recovered and multiplied extensively. Those extinction events were caused by catastrophic alterations of the environment, such as massive volcanic eruptions, depletion of oceanic oxygen, or collision with an asteroid (5). In each case, it took millions of years to regain numbers of species comparable to those that existed before the particular extinction event (6, 7). Even though only an estimated 2% of all of the species that ever lived are alive today, the absolute number of species is greater now than ever before (2). It was into such a biologically diverse world that we humans evolved, and such a world that we are destroying.

Life has now entered a sixth mass extinction (8–10). This is probably the most serious environmental problem, because the loss of a species is permanent, each of them playing a greater or lesser role in the living systems on which we all depend (11, 12). The species extinctions that define the current crisis are, in turn, based on the massive disappearance of their component populations, mostly since the 1800s (10, 13–20). The massive losses that we are experiencing are being caused, directly or indirectly, by the activities of *Homo sapiens.* They have almost all occurred since our ancestors developed agriculture, some 11,000 y ago. At that time, we numbered about 1 million people worldwide; now there are 7.7 billion of us, and our numbers are still rapidly growing (21). As our numbers have grown, humanity has come to pose an unprecedented threat to the vast majority of its living companions.

Today, species extinction rates are hundreds or thousands of times faster than the “normal” or “background” rates prevailing in the last tens of millions of years (8–10). The recent United Nations report on biodiversity and ecosystem services estimates that a quarter of all species face extinction, many within decades (11). When a species disappears, a wide range of characteristics is lost forever, from genes and interactions to phenotypes and behaviors (22–27).

Every time a species or population vanishes, Earth’s capability to maintain ecosystem services is eroded to a degree, depending on the species or population concerned. Each population is likely to be unique and therefore likely to differ in its capacity to fit into a particular ecosystem and play a role there. The effects of extinctions will worsen in the coming decades, as losses of functional units, redundancy, and genetic and cultural variability change entire ecosystems (14, 23, 24). Humanity needs the life support of a relatively stable climate, flows of fresh water, agricultural pest and disease-vector control, pollination for crops, and so on, all provided by functional ecosystems (12, 28).

Examples documenting the ongoing biological annihilation are proliferating, each of them underlining the magnitude of the problem and the urgency of taking action. More than 400 vertebrate species became extinct in the last 100 y, extinctions that would have taken up to 10,000 y in the normal course of evolution (10). Among vertebrate species that have disappeared in historic times are the thylacine (*Thylancinus cyanocephalus*), the ivory-billed woodpecker (*Campephilus principalis)*, and the Round Island burrowing boa (*Bolyeria multocarinata*). Champions of recent extinctions are amphibians, with hundreds of species of frogs and toads suffering population declines and extinctions: perhaps a fifth of the species extinct already or on the brink of extinction. The symbol of this amphibian holocaust is the loss, soon after it was discovered, of the gorgeous golden toad (*Incilius periglenes*), an inhabitant of Costa Rican cloud forests. The principal culprit in the disappearance of so many amphibians so rapidly is a chytrid fungus, sometimes spread from place to place as a result of human activities (29); this parasite affects populations weakened by climate disruption particularly rapidly (30).

Millions of populations have vanished in the last 100 y, with most people unaware of their loss (16); such losses have become extremely severe in the last few decades (13–20, 31, 32). These losses are not simply happening to obscure organisms of little interest to anyone. Instead, they include many populations of large and conspicuous animals and plants, from lions and tigers to elephants and cacti. For example, in a sample of 177 species of large mammals, most lost more than 80% of their geographic range in the last century (13), implying a very extensive extirpation of populations. Similarly, a recent study showed that 32% of more than 27,000 vertebrate species have declining populations (15). And the Living Planet Report found that roughly 70% of all individuals of vertebrate species have disappeared over the 50 y since 1970 (33). Insects and other invertebrates have suffered huge losses also. About 75% of all flying insects in national parks in Germany disappeared in 25 y (16), and there are numerous signs that many species of insects are heading for the exit (34, 35). Similar losses have been documented for various species of clams, snails, and starfish (36–40) and for plants (19, 41). The process of extinction involves progressive declines in the abundance and geographic range of a species (26). Smaller populations become more isolated and more prone to extinction from natural (e.g., inbreeding, accident) and human causes (42). The reason so many species are being pushed to extinction by anthropogenic causes is indicated by humans and their domesticated animals being some 30 times the living mass of all of the wild mammals that must compete with them for space and resources (43).

When the number of individuals in a population or species drops too low, its contributions to ecosystem functions and services become unimportant, its genetic variability and resilience is reduced, and its contribution to human welfare may be lost. At a certain point, a population can be too small or too lacking in required habitat to reproduce itself. At one time, the bison (*Bison bison*) was a keystone species in the prairies of North America, maintaining the entire ecosystem, supplying, at various stages, meat, robes, and fertilizer to Native Americans, and later to Europeans. Indeed, it is estimated that, two centuries ago, there were probably some 30 to 60 million of these large mammals roaming the plains of the continent. Overharvesting for meat and skins, and prairie ecosystems converted to farming, exterminated most populations. By 1884, only some 325 individuals were left (44). Subsequently, they have recovered to about 4,000 wild bison, with some 500,000 living in enclosures; the species has certainly not reclaimed its ecological role; in any case, the great majority of North American prairies have been destroyed. In a sense, bison and many other species with tiny populations have become what Janzen (45) has termed, in a slightly different way, “ecological zombies,” still there but not significant for ecosystem function.

**Fig. 1.**
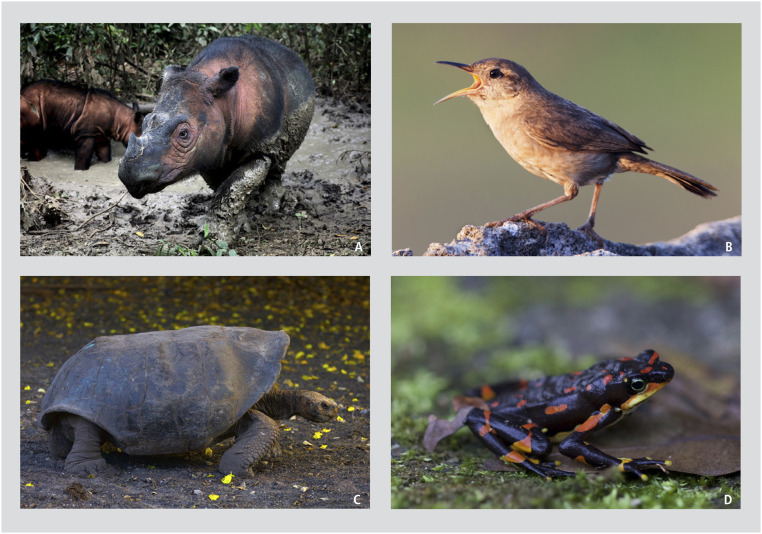
Terrestrial vertebrates on the brink (i.e., with 1,000 or fewer individuals) include species such as (*A*) Sumatran rhino (*Dicerorhinus sumatrensis*; image credit: Rhett A. Butler [photographer]), (*B*) Clarion island wren (*Troglodytes tanneri*; image credit: Claudio Contreras Koob [photographer]), (*C*) Española Giant Tortoise (*Chelonoidis hoodensis*; image credit: G.C.), and (*D*) Harlequin frog (*Atelopus varius*; the population size of the species is unknown but it is estimated at less than 1,000; image credit: G.C.).

Here we add to our studies of the sixth mass extinction (9, 10, 13–16, 42). We analyze the status of vertebrates that we judge are on the brink of extinction. Most of these have lost the majority of their geographic range, and most of their populations, and now have fewer than 1,000 individuals (referred to as “on the brink” or “under 1,000” hereafter). Examples of those species are shown in Fig. 1. We then compare the distribution patterns of these species on the brink with those slightly more secure—species estimated to have more than 1,000 but less than 5,000 individuals (referred as “under 5,000” hereafter). We chose terrestrial vertebrates because they are the animals most familiar to people and because there are more data on their conservation status than on those of most other organisms. And we pick, as a center of discussion, a round number of individuals, 1,000, which is the population size at which the International Union for Conservation of Nature (IUCN) ordinarily lists species as “critically endangered.” So, we specifically address the following questions about the species on the brink: 1) Which are the vertebrate species on the brink? 2) What are the historic and current patterns of distribution of those species? 3) How many populations have they lost in historic times? 4) How do these patterns compare with those of the slightly more secure species—those under 5,000 individuals? The answers to these questions should allow us, in principle, to take effective action to save the species concerned from extinction, but, to succeed, our efforts must be prompt, determined, and widespread. We need to undertake such efforts to have any chance of reversing the biological annihilation that is underway.

## Results

### Vertebrates on the Brink of Extinction.

**Fig. 2.**
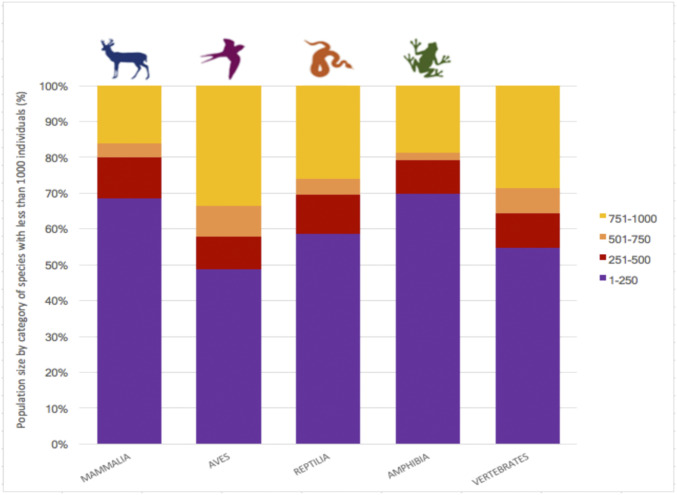
Population size of terrestrial vertebrate species on the brink (i.e., with under 1,000 individuals). Most of these species are especially close to extinction because they consist of fewer than 250 individuals. In most cases, those few individuals are scattered through several small populations.

Our results underline the magnitude of the extinction crises at both species and population levels. Although the data are necessarily incomplete, at least 1.7% of the species of terrestrial vertebrates—515 of them—have fewer than 1,000 remaining individuals, that is, are on the brink (Table 1 and Fig. 2). Those 515 species represent, however, a staggering quarter of the species, both common and rare, that have population data. Among these under on the brink, 243 (47%) are continental and 272 (53%) are insular. Most of the species are from South America (157 species [spp], 30%), followed by Oceania (108 spp, 21%), Asia (106 spp, 21%), Africa (82 spp, 16%), North and Central America (55 spp, 11%), and Europe (6 spp, 1%). The greatest numbers of mammals on the brink occur in Asia and Oceania, while most birds on the brink live in South America and Oceania (Table 2). The reptiles with very small populations occur mainly in North America and Asia, while the amphibians are in the Americas. Proportionally, more bird species are on the brink, followed by amphibians, then mammals, and reptiles.

**Table 1.** Number of species on the brink (i.e., with fewer than 1,000 individuals) and number of species whose conservation status had been evaluated by the IUCN (Version 2019)

Class	Species	IUCN	Percent
Mammalia	74	5,459	1.4
Aves	335	10,423	3.2
Reptilia	41	6,861	0.6
Amphibia	65	6,631	1.0
Total	515	29,374	1.7

**Table 2.** Distribution of species on the brink (i.e., with fewer than 1,000 individuals) across continents

Class	Africa	Asia	Europe	South America	North America	Oceania
Mammalia	13	22	2	8	12	17
Aves	64	67	4	86	28	85
Reptilia	2	12	0	10	12	5
Amphibia	3	5	0	53	3	1
Total	82	106	6	157	55	108

**Fig. 3.**
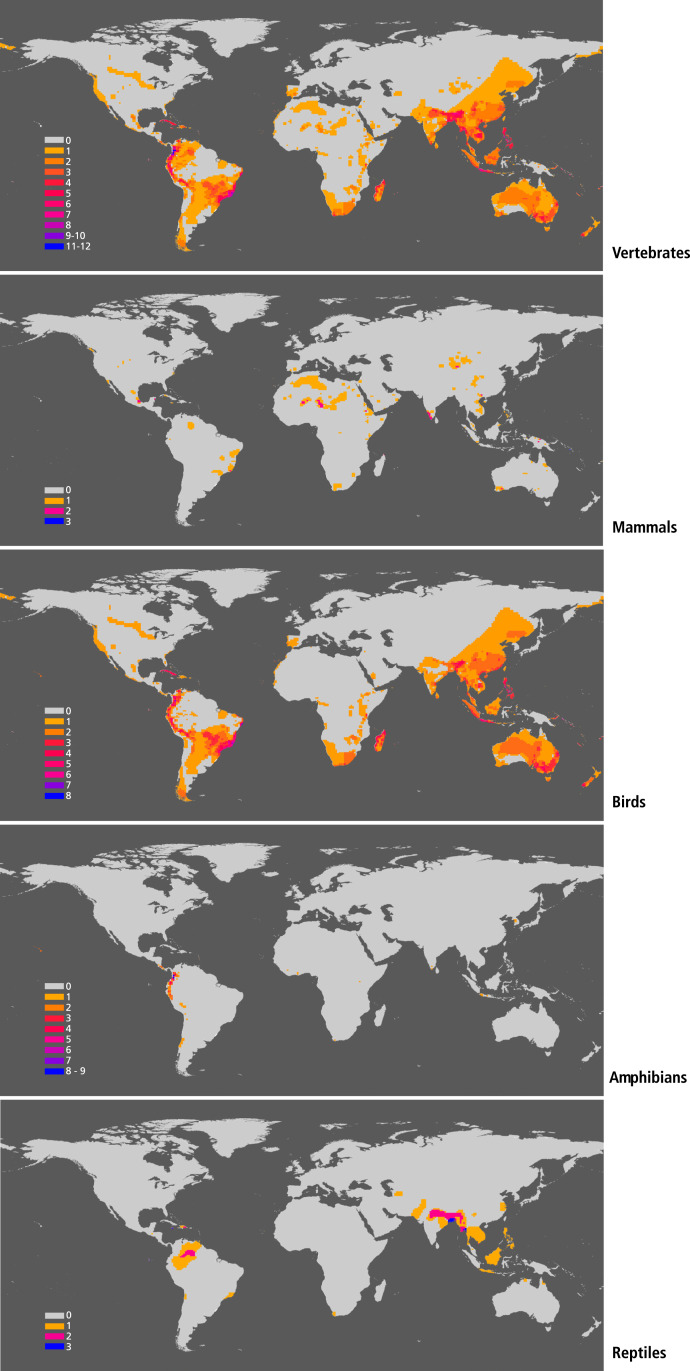
Geographic distribution terrestrial vertebrate species on the brink (i.e., with under 1,000 individuals). The colors in the left bar indicate the number of species in a 100 square km global cell grid.

Population sizes of the species on the brink are often much smaller than 1,000 individuals (Fig. 2). Indeed, more than half of these species are estimated to have been reduced to 250 or fewer individuals (Fig. 2). The number of species with 250 or fewer individual in mammals and amphibians is even higher, with roughly two-thirds in this category. Species on the brink are concentrated, not surprisingly, in areas highly impacted by humans (Fig. 3). Different groups display diverse geographic patterns. Bird species on the brink are more widespread than those of other vertebrates (Fig. 3). But the majority of all vertebrates on the brink are found in tropical and subtropical regions in the Americas, Africa, and Asia. Those are areas with relatively rich biotas, with many endemic species, and smaller populations, on the average, than the species of the vast north temperate regions of the globe.

### The Road to the Brink: From 5,000 to 1,000 Individuals.

**Fig. 4.**
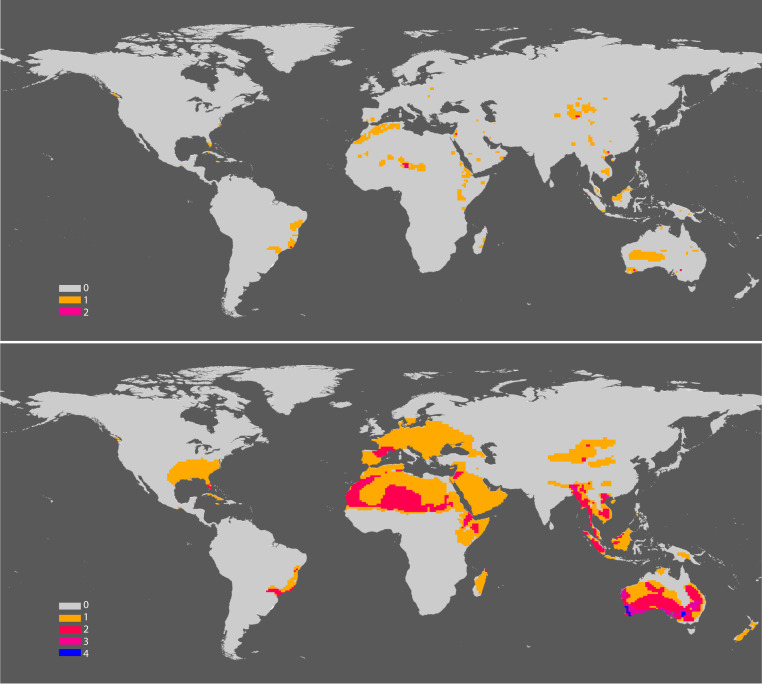
Historic (*Top*) and current (*Bottom*) geographic range of 48 mammal and 29 bird terrestrial species on the brink (i.e., with under 1,000 individuals). Note the high concentration of species in tropical regions throughout the world.

The distributions of the 388 species comprising the under 5,000s and of those at the brink (i.e., under 1,000s) show a near-universal distribution, the main exceptions being temperate and subarctic regions of the Northern Hemisphere (Fig. 4). Those species are concentrated largely in tropical regions. The distribution of those on the brink and the under 5,000s shows a significant concurrence; an impressive 84% of the under 5,000s species are found in the same regions as the species on the brink. The congruence is further evidence that the present sixth mass extinction is human caused, something further indicated by what seems to be an incipient regional biodiversity collapse in those areas. As population extinctions continue, some of the species on the brink will likely become extinct, and some of the under 5,000s will move onto the brink.

### Mass Extinction of Populations: Comparing Historic and Current Range.

**Fig. 5.**
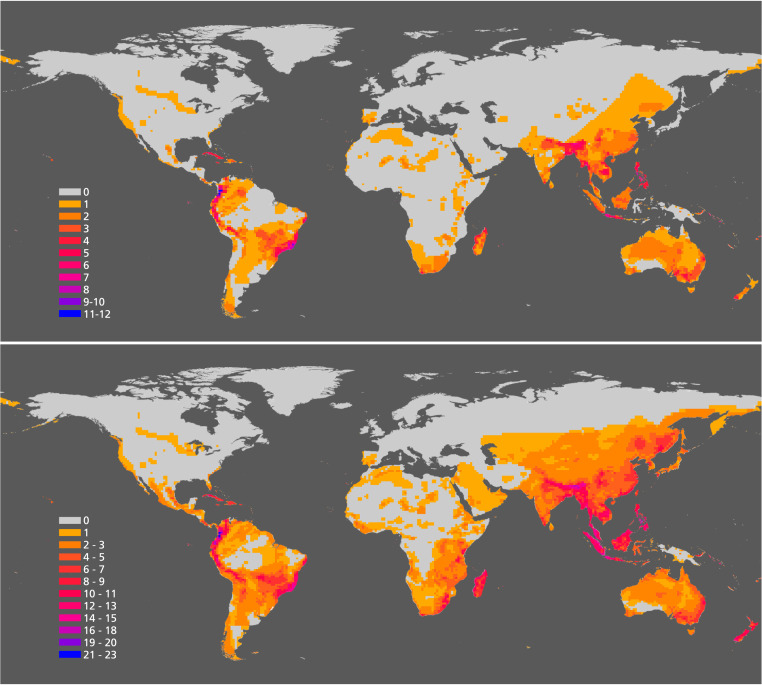
Current distribution of 515 terrestrial vertebrate species on the brink (i.e., with under 1,000 individuals; *Top*) and 903 species with under 5,000 individuals (*Bottom*). Of the 388 species under 5,000 that have populations larger than 1,000 individuals, 84% have overlapping distributions with the species at the brink (i.e., with under 1,000 individuals), indicating high distribution congruence.

Species at the brink have lost most of their populations and individuals. To gain insights into the extent and significance of population extinction, we compared the historic and current distributions of both 48 species of mammals and 29 species of birds on the brink; mammals and birds were the only groups for which such data were available (Fig. 5). These comparisons show a huge reduction of the historic geographic range of those species, representing a massive loss of populations too. Vast areas in Europe, northern Africa, the Middle East, Australia, and North America have lost most of those mammals and birds that are now on the brink. Assuming that an average mammal or bird population occupies 10,000 km^2^ (13), our data suggest that, during the last two centuries, of the 48 mammal and of the 29 bird species we examined, roughly 3,600 populations of the 48 mammal species and 2,930 populations of the 29 bird species have disappeared. Those mammal and bird species have lost an average 95% and 94% of their geographic range since 1900. If we assume a similar reduction of the historic range of all of the 515 vertebrate species on the brink, then a staggering 237,000 populations of their populations have disappeared since 1900.

### Mass Extinction of Species: Accelerated Human-Induced Rates.

The species on the brink could soon be joining the ∼543 species of vertebrates that are known to have disappeared since 1900 (46). Suppose, as a thought experiment, that the species at the brink will become extinct by the year 2050. Then the sum of 543 already extinct and those on the brink projected to disappear would be 1,058 vertebrates. Under the last 2 million years’ background rate, 2 species would be expected to become extinct in a century for every 10,000 species (8). Therefore, for the 29,400 vertebrate species evaluated in our study, one would expect 9 extinctions in the 150 y between 1900 and 2050. Instead of the 9 expected extinctions in the hypothetical scenario, 1,058 species would become extinct by 2050. So, the extinction rate by 2050 would be 117 times higher than the background rate. Therefore, the species extinct in those 150 y would have taken 11,700 y to become extinct under the background extinction rate.

## Discussion

Our results provide important conservation insights. First, as the status of any species changes from common to being on the brink of extinction, its gradual loss will bring pressures to bear on other species with which it interacts. Clearly, the loss of so many populations has caused major changes in the ecosystems they inhabit and doubtless contributed concurrently to the extinction of other species in those ecosystems. At times, these losses must have triggered the substantial alteration of ecosystems structure and function (11, 12). Extinction cascades, a series of extinctions triggered by the disappearance of a keystone species in an ecosystem, occur frequently, as shown by the classic case of Steller’s sea cow (47). Obviously, the loss of species and populations drives the loss of their specialized parasites. A dramatic example was the discovery and description in 2005 of six new species of mites found among the feathers of museum specimens of the long extinct Carolina Parakeet (*Conuropsis carolinensis*) (48). Similarly, closely linked pairs of species involved in relationships like pollination may become extinct simultaneously (49–51). Thus, moving onto the brink is an important part of the process of defaunation (14).

Second, documenting so many species on the verge of collapse has led us to suggest that future rates of extinction are probably underestimated. As our results indicate, the current rapid vertebrate extinction rate will itself increase sharply in the future. So, predictions that one-fifth of all species would be in danger of extinction by midcentury and half or more by the end of the century begin to make sense (9–11).

Third, concentrations of species at risk for extinction, with the geographical congruence in distribution of the under 5,000s and species on the brink categories, are strong indicators of incipient regional biodiversity collapse in areas such as the Arctic, southeast Asia, and elsewhere. They occur mostly in heavily populated regions such as tropical Asia, where major ongoing biodiversity losses are well known (10, 13–15, 30, 46, 52, 53).

Fourth, species at the brink have been pushed to a critical conservation status because of human activities, where habitat loss and fragmentation, illegal trade, overexploitation, introduced domestic and wild species, toxification, and pollution have played a major role (10, 13–15, 30, 46, 52, 53). More recently, climate disruption is becoming a major cause of species endangerment (10, 11, 14–17). We believe that the recent coronavirus outbreak is linked to wildlife trade and consumption in China (54). The ban on wildlife trade imposed by the Chinese government could be a major conservation measure for many species on the brink, if imposed properly. The ban should include wild species for consumption as food as well as medicinal use and pets.

Finally, major losses of populations and species clearly impede the provision of ecosystem goods and services, with consequent impacts on human well-being (11, 12). The growing human population, increasing rates of consumption, and projected growth in the future can only accelerate the rapid disappearance of species, now a stream, to a rushing torrent—a problem for survival that only human beings have the power to alleviate.

## Concluding Remarks

The extinction crisis, like the toxification and climate crises to which it is tied, poses an existential threat to civilization. Although it is more immediate than climate disruption, its magnitude and likely impacts on human well-being are largely unknown by governments, the private sector, and civil society. It is, therefore, a scientific and moral imperative for scientists to take whatever actions they can to stop extinction. For example, in relation to all under 5,000s species, they should be immediately classified by the IUCN as critically endangered. Indeed, the conservation of endangered species should be elevated to a national and global emergency for governments and institutions, equal to climate disruption. Among the possible actions, a global comprehensive binding agreement is required to address the extinction crisis, especially to tackle the legal and illegal trade in wild species. Such an agreement should be a mere first step in developing a 2020–2030 conservation agenda.

Many of the species endangered or at the brink of extinction are being decimated by the legal and illegal wildlife trade, which poses a fundamental threat for human health and well-being, is a major cause of population and species extinctions, and is eroding the ecosystem services that we require to survive. The horrific coronavirus disease 2019 (Covid-19) pandemic that we are experiencing, of which we still do not fully understand the likely economic, political, and social global impacts, is linked to wildlife trade. It is imperative that wildlife trade for human consumption is considered a gigantic threat to both human health and wildlife conservation. Therefore, it has to be completely banned, and the ban strictly enforced, especially in China, Vietnam, Indonesia, and other countries in Asia (14, 42, 54). It is also imperative that steps are taken to provide food for the poor people that conservation measures may deprive of bush meat, especially in Africa (42).

In view of the current extinction crisis and the lack of widespread actions to halt it, it is very important that scientists should metaphorically take to the streets. We have, for example, started a new global initiative we called “Stop Extinction,” to address and publicize the extent of the extinction crisis and its impacts on the loss of biodiversity, ecosystem services, and human well-being, aspects still rather ignored by most people (55). There is time, but the window of opportunity is almost closed. We must save what we can, or lose the opportunity to do so forever. There is no doubt, for example, that there will be more pandemics if we continue destroying habitats and trading wildlife for human consumption as food and traditional medicines. It is something that humanity cannot permit, as it may be a tipping point for the collapse of civilization. What is at stake is the fate of humanity and most living species. Future generations deserve better from us.

## Methods

In order to obtain size-based population of global terrestrial vertebrate’s information, we acquired database material from the IUCN Red List of Threatened Species and Birdlife International (46, 56). We assembled data on the geographical range of each species, and then classified them within one of the nine categories of the IUCN Red List (46). On the basis of these data, we determined the number of vertebrate species with a maximum population size of 1,000 individuals. According to the IUCN, the number of individuals is based on the population size measured as the number of mature individuals. We excluded extinct species from our analyses, leaving a total of 515 species, out of which 65 are amphibians, 335 are birds, 41 are reptiles, and 74 are terrestrial mammals (*SI Appendix*). Distribution ranges of all groups were overlapped and combined to obtain the global maps of number of species (richness) using ARCGIS 10.1. This involved using a Behrmann equal‐area grid with a cell size of 96.5 km × 96.5 km (∼1° at the equator).

The population extinction analysis was conducted with 48 species of terrestrial mammals and 29 bird species distributed on five continents. The historical distribution was gathered from specialized literature (13), and the spatial data of current distribution were obtained from the IUCN Red List (46) (*SI Appendix*). The historical and current distribution ranges were overlapped to obtain the global maps of number of species using ARCGIS 10.1. For the 48 terrestrial mammal and 29 bird species, we calculated the area of the historical and current geographic distribution ranges to estimate the lost area and the area where species currently are distributed (in square kilometers).

### Data Availability.

All data are available within the manuscript and in *SI Appendix*.

## Supplementary Material

Supplementary File
