# Supplementary material for: Vertebrates on the brink as indicators of biological annihilation and the sixth mass extinction
Source: Proc Natl Acad Sci U S A. 2020 Jun 1;117(24):13596–602. doi: 10.1073/pnas.1922686117 (PMC7306750; doi:10.1073/pnas.1922686117)
Supplement: Supplementary File [file pnas.1922686117.sapp.pdf]

VERTEBRATE SPECIES WITH LESS THAN 1000 INDIVIDUALS (IUCN, 2019)

SCIENTIFIC NAME	COMMON NAME	Category
**MAMMALS**		
Addax nasomaculatus	Addax	CR
Ailuropoda melanoleuca	Giant Panda	VU
Aproteles bulmerae	Bulmer's Fruit Bat	CR
Axis kuhlii	Bawean Deer	CR
Babyrousa togeanensis	Togian Islands Babirusa	EN
Beatragus hunteri	Hirola	CR
Bos sauveli	Kouprey	CR
Brachyteles hypoxanthus	Northern Muriqui	CR
Bubalus mindorensis	Tamaraw	CR
Bunolagus monticularis	Riverine Rabbit	CR
Caenolestes condorensis	Andean Caenolestid	VU
Callicebus barbarabrownae	Blond Titi Monkey	CR
Camelus ferus	Bactrian Camel	CR
Canis rufus	Red Wolf	CR
Canis simensis	Ethiopian Wolf	EN
Capra walie	Walia Ibex	EN
Cavia intermedia	Santa Catarina’s Guinea Pig	CR
Cercopithecus dryas	Dryad Monkey	CR
Coleura seychellensis	Seychelles Sheath-tailed Bat	CR
Crocidura trichura	Christmas Island Shrew	CR
Dendrolagus mayri	Wondiwoi Tree-kangaroo	CR
Dendrolagus pulcherrimus	Golden-mantled Tree Kangaroo	CR
Dendrolagus scottae	Tenkile	CR
Dicerorhinus sumatrensis	Sumatran Rhinoceros	CR
Dipodomys gravipes	San Quintin Kangaroo Rat	CR
Equus africanus	African Wild Ass	CR
Equus ferus	Przewalski's Horse	EN
Gazella leptoceros	Slender-horned Gazelle	EN
Habromys simulatus	Jico Deer Mouse	CR
Hipposideros hypophyllus	Kolar Leaf-nosed Bat	CR
Lasiorhinus krefftii	Northern Hairy-nosed Wombat	CR
Leontopithecus chrysopygus	Black Lion Tamarin	EN
Leontopithecus rosalia	Golden Lion Tamarin	EN
Lepilemur septentrionalis	Sahafary Sportive Lemur	CR
Lonchophylla dekeyseri	Dekeyser's Nectar Bat	EN
Lonchorhina fernandezi	Fernandez's Sword-nosed Bat	EN
Lynx pardinus	Iberian Lynx	EN
Marmota vancouverensis	Vancouver Island Marmot	CR
Martes gwatkinsii	Nilgiri Marten	VU
Mesocapromys nanus	Dwarf Hutia	CR

www.pnas.org/cgi/doi/10.1073/pnas.1922686117

Mesocapromys sanfelipensis	Little Earth Hutia	CR
Mirimiri acrodonta	Fijian Monkey-faced Bat	CR
Mustela nigripes	Black-footed Ferret	EN
Myotis planiceps	Flat-headed Myotis	EN
Myrmecobius fasciatus	Numbat	EN
Nanger dama	Dama Gazelle	CR
Nomascus hainanus	Hainan Gibbon	CR
Nomascus nasutus	Cao-vit Crested Gibbon	CR
Oryx leucoryx	Arabian Oryx	VU
Parantechinus apicalis	Dibbler	EN
Peromyscus mekisturus	Puebla Deer Mouse	CR
Petrogale sharmani	Mount Claro Rock Wallaby	VU
Phalanger matanim	Telefomin Cuscus	CR
Phyllonycteris aphylla	Jamaican Flower Bat	CR
Pipistrellus maderensis	Madeira Pipistrelle	VU
Porcula salvania	Pygmy Hog	CR
Potorous gilbertii	Gilbert's Potoroo	CR
Procyon pygmaeus	Pygmy Raccoon	CR
Propithecus perrieri	Perrier’s Sifaka	CR
Pseudoryx nghetinhensis	Saola	CR
Pteralopex pulchra	Montane Monkey-faced Bat	CR
Pteropus pselaphon	Bonin Flying Fox	EN
Reithrodontomys spectabilis	Cozumel Harvest Mouse	CR
Rhinoceros sondaicus	Javan Rhinoceros	CR
Rhinolophus cohenae	Cohen's Horseshoe Bat	VU
Rhinopithecus strykeri	Myanmar Snub-nosed Monkey	CR
Rhinopoma hadramauticum	Yemeni Mouse-tailed Bat	EN
Rusa alfredi	Philippine Spotted Deer	EN
Trachypithecus poliocephalus	White-headed Langur	CR
Uromys imperator	Emperor Rat	CR
Uromys porculus	Guadalcanal Rat	CR
Viverra civettina	Malabar Civet	CR
Zyzomys palatalis	Carpentarian Rock-rat	CR
Zyzomys pedunculatus	Central Rock-rat	CR
**BIRDS**		
Accipiter gundlachi	Gundlach's Hawk	EN
Accipiter imitator	Imitator Goshawk	VU
Acridotheres melanopterus	Black-winged Myna	CR
Acridotheres tertius	Grey-rumped Myna	CR
Acridotheres tricolor	Grey-backed Myna	CR
Acrocephalus caffer	Tahiti Reed-warbler	EN
Acrocephalus familiaris	Millerbird	CR

Acrocephalus sorghophilus	Streaked Reed-warbler	EN
Acrocephalus vaughani	Pitcairn Reed-warbler	EN
Actenoides bougainvillei	Bougainville Moustached Kingfisher	EN
Aepypodius bruijnii	Waigeo Brush-turkey	EN
Aerodramus sawtelli	Atiu Swiftlet	VU
Agelaius xanthomus	Yellow-shouldered Blackbird	EN
Alauda razae	Raso Lark	CR
Alcedo euryzona	Javan Blue-banded Kingfisher	CR
Alopecoenas canifrons	Palau Ground-dove	EN
Alopecoenas erythropterus	Polynesian Ground-dove	CR
Alopecoenas kubaryi	Caroline Ground-dove	VU
Alopecoenas rubescens	Marquesas Ground-dove	EN
Amaurocichla bocagii	Sao Tome Short-tail	VU
Amazilia luciae	Honduran Emerald	EN
Amazona arausiaca	Red-necked Amazon	VU
Amazona guildingii	St Vincent Amazon	VU
Amazona imperialis	Imperial Amazon	EN
Amazona versicolor	St Lucia Amazon	VU
Anairetes alpinus	Ash-breasted Tit-tyrant	EN
Anas albogularis	Andaman Teal	VU
Anas laysanensis	Laysan Duck	CR
Anas nesiotis	Campbell Teal	EN
Anodorhynchus leari	Lear's Macaw	EN
Anthochaera phrygia	Regent Honeyeater	CR
Antilophia bokermanni	Araripe Manakin	CR
Apalis fuscigularis	Taita Apalis	CR
Aphrastura masafucrae	Masafuera Rayadito	CR
Aplonis santovestris	Santo Starling	EN
Apteryx rowi	Okarito Kiwi	VU
Apus acuticauda	Dark-rumped Swift	VU
Aquila adalberti	Spanish Imperial Eagle	VU
Ara glaucogularis	Blue-throated Macaw	CR
Ara rubrogenys	Red-fronted Macaw	CR
Ardea humbloti	Madagascar Heron	EN
Ardea insignis	White-bellied Heron	CR
Ardeotis nigriceps	Great Indian Bustard	CR
Artisornis moreaui	Long-billed Tailorbird	CR
Artisornis sousae	Mozambique Forest-warbler	EN
Asarcornis scutulata	White-winged Duck	EN
Asthenes perijana	Perija Thistletail	EN

Atlapetes flaviceps	Yellow-headed Brush-finch	EN
Atlapetes pallidiceps	Pale-headed Brush-finch	EN
Aythya baeri	Baer's Pochard	CR
Aythya innotata	Madagascar Pochard	CR
Bostrychia bocagei	Dwarf Ibis	CR
Branta sandvicensis	Hawaiian Goose	VU
Buteo galapagoensis	Galapagos Hawk	VU
Buteo ridgwayi	Ridgway's Hawk	CR
Buteo socotraensis	Socotra Buzzard	VU
Buteo ventralis	Rufous-tailed Hawk	VU
Buteogallus coronatus	Crowned Solitary Eagle	EN
Cacatua haematuropygia	Philippine Cockatoo	CR
Calicalicus rufocarpalis	Red-shouldered Vanga	VU
Calidris pygmaea	Spoon-billed Sandpiper	CR
Capito wallacei	Scarlet-banded Barbet	VU
Carpococcyx viridis	Sumatran Ground-cuckoo	CR
Carpodectes antoniae	Yellow-billed Cotinga	EN
Centropus steerii	Black-hooded Coucal	CR
Certhia tianquanensis	Sichuan Treecreeper	NT
Ceyx sangirensis	Sangihe Dwarf-kingfisher	CR
Chaetocercus berlepschi	Esmeraldas Woodstar	EN
Charadrius obscurus	Southern Red-breasted Plover	CR
Charadrius sanctaehelenae	St Helena Plover	VU
Charmosyna toxopei	Blue-fronted Lorikeet	CR
Chlorophoneus kupeensis	Mount Kupe Bush-shrike	EN
Chondrohierax wilsonii	Cuban Kite	CR
Chrysocolaptes xanthocephalus	Yellow-faced Flameback	EN
Ciconia stormi	Storm's Stork	EN
Cinclodes aricomae	Royal Cinclodes	CR
Cinclodes palliatus	White-bellied Cinclodes	CR
Cinnyris rockefelleri	Rockefeller's Sunbird	VU
Circus macrosceles	Madagascar Marsh-harrier	EN
Circus maillardi	Reunion Marsh-harrier	EN
Circus maurus	Black Harrier	EN
Cissa thalassina	Javan Green Magpie	CR
Claravis geoffroyi	Purple-winged Ground-dove	CR
Clytoctantes alixii	Recurve-billed Bushbird	EN
Clytorhynchus sanctaecrucis	Nendo Shrikebill	EN
Coccyzus ferrugineus	Cocos Cuckoo	VU
Coeligena orina	Glittering Starfrontlet	CR

Colaptes fernandinae	Fernandina's Flicker	VU
Columba thomensis	Sao Tome Olive-pigeon	EN
Columbina cyanopis	Blue-eyed Ground-dove	CR
Conothraupis mesoleuca	Cone-billed Tanager	EN
Copsychus sechellarum	Seychelles Magpie-robin	EN
Coracopsis barklyi	Seychelles Parrot	VU
Coracornis sanghirensis	Sangihe Whistler	CR
Corvus kubaryi	Mariana Crow	CR
Corvus unicolor	Banggai Crow	CR
Cotinga maculata	Banded Cotinga	EN
Crax blumenbachii	Red-billed Curassow	EN
Crax globulosa	Wattled Curassow	EN
Crithagra concolor	Sao Tome Grosbeak	CR
Crithagra flavigula	Yellow-throated Seedeater	EN
Cyanolimnas cerverai	Zapata Rail	CR
Cyanoramphus forbesi	Chatham Parakeet	VU
Cyanoramphus malherbi	Malherbe's Parakeet	CR
Cyclopsitta coxeni	Coxen's Fig-parrot	EN
Dasyornis longirostris	Western Bristlebird	EN
Dendrocitta bayleii	Andaman Treepie	VU
Dendrocopos noguchii	Okinawa Woodpecker	CR
Dicaeum quadricolor	Cebu Flowerpecker	CR
Dicrurus aldabranus	Aldabra Drongo	NT
Dicrurus fuscipennis	Grand Comoro Drongo	EN
Dicrurus menagei	Tablas Drongo	EN
Didunculus strigirostris	Tooth-billed Pigeon	CR
Diomedea amsterdamensis	Amsterdam Albatross	EN
Dreptes thomensis	Giant Sunbird	VU
Ducula galeata	Nukuhiva Imperial-pigeon	EN
Edolisoma nesiotis	Yap Cicadabird	EN
Elaenia ridleyana	Noronha Elaenia	VU
Eleoscytalopus psychopompus	Bahia Tapaculo	EN
Emberiza jankowskii	Rufous-backed Bunting	EN
Eriocnemis isabellae	Gorgeted Puffleg	CR
Eriocnemis mirabilis	Colorful Puffleg	EN
Eriocnemis nigrivestis	Black-breasted Puffleg	CR
Erythropitta caeruleitorques	Sangihe Pitta	EN
Erythropitta palliceps	Siau Pitta	EN
Erythropitta splendida	Tabar Pitta	VU
Eulidia yarrellii	Chilean Woodstar	CR

Eutrichomyias rowleyi	Cerulean Paradise-flycatcher	CR
Eutriorchis astur	Madagascar Serpent-eagle	EN
Falco araeus	Seychelles Kestrel	VU
Falco fasciinucha	Taita Falcon	VU
Falco hypoleucos	Grey Falcon	VU
Falco punctatus	Mauritius Kestrel	EN
Formicivora paludicola	Marsh Antwren	CR
Forpus xanthops	Yellow-faced Parrotlet	VU
Foudia rubra	Mauritius Fody	EN
Fringilla polatzeki	Gran Canaria Blue Chaffinch	EN
Gallicolumba keayi	Negros Bleeding-heart	CR
Gallicolumba platenae	Mindoro Bleeding-heart	CR
Garrulax courtoisi	Blue-crowned Laughingthrush	CR
Garrulax rufifrons	Rufous-fronted Laughingthrush	CR
Geospiza acutirostris	Genovesa Ground-finch	VU
Geospiza conirostris	Espanola Cactus-finch	VU
Geospiza heliobates	Mangrove Finch	CR
Geospiza propinqua	Genovesa Cactus-finch	VU
Geospiza septentrionalis	Vampire Ground-finch	VU
Geronticus eremita	Northern Bald Ibis	EN
Glaucis dohrnii	Hook-billed Hermit	EN
Gorsachius magnificus	White-eared Night-heron	EN
Gracula robusta	Nias Hill Myna	CR
Grallaria fenwickorum	Antioquia Antpitta	CR
Grallaria kaestneri	Cundinamarca Antpitta	EN
Grallaria ridgelyi	Jocotoco Antpitta	EN
Grallaricula ochraceifrons	Ochre-fronted Antpitta	EN
Grus americana	Whooping Crane	EN
Gymnogyps californianus	California Condor	CR
Gymnomyza aubryana	Crow Honeyeater	CR
Gymnomyza samoensis	Mao	EN
Haematopus chathamensis	Chatham Oystercatcher	EN
Haliaeetus sanfordi	Sanford's Sea-eagle	VU
Haliaeetus vociferoides	Madagascar Fish-eagle	CR
Hapalopsittaca fuertesi	Indigo-winged Parrot	CR
Hemignathus wilsoni	Akiapolaau	EN
Hemiphaga chathamensis	Chatham Pigeon	VU
Herpsilochmus parkeri	Ash-throated Antwren	EN
Heteroglaux blewitti	Forest Owlet	EN
Heteromirafra archeri	Liben Lark	CR

Houbaropsis bengalensis	Bengal Florican	CR
Hydrobates monteiroi	Monteiro's Storm-petrel	VU
Hypotaenidia okinawae	Okinawa Rail	EN
Hypotaenidia sylvestris	Lord Howe Woodhen	EN
Hypsipetes olivaceus	Mauritius Black Bulbul	VU
Icterus northropi	Bahama Oriole	CR
Icterus oberi	Montserrat Oriole	VU
Jacamaralcyon tridactyla	Three-toed Jacamar	VU
Junco insularis	Guadalupe Junco	EN
Lalage newtoni	Reunion Cuckooshrike	CR
Lalage typica	Mauritius Cuckooshrike	VU
Laniarius amboimensis	Gabela Bush-shrike	EN
Laniarius brauni	Orange-breasted Bush-shrike	EN
Lanius newtoni	Sao Tome Fiscal	CR
Larus fuliginosus	Lava Gull	VU
Lepidopyga lilliae	Sapphire-bellied Hummingbird	CR
Leptasthenura xenothorax	White-browed Tit-spinetail	EN
Leptodon forbesi	White-collared Kite	EN
Leptotila wellsi	Grenada Dove	CR
Leucocarbo carunculatus	Rough-faced Shag	VU
Leucocarbo onslowi	Chatham Shag	CR
Leucocarbo ranfurlyi	Bounty Shag	VU
Linaria johannis	Warsangli Linnet	EN
Liocichla bugunorum	Bugun Liocichla	CR
Lipaugus weberi	Chestnut-capped Piha	CR
Loddigesia mirabilis	Marvelous Spatuletail	EN
Lophornis brachylophus	Short-crested Coquette	CR
Lophura edwardsi	Edwards's Pheasant	CR
Loxops caeruleirostris	Akekee	CR
Manorina melanotis	Black-eared Miner	EN
Megalurulus grosvenori	Bismarck Thicketbird	VU
Megalurulus rufus	Long-legged Thicketbird	EN
Megapodius pritchardii	Tongan Scrubfowl	EN
Melanospiza richardsoni	St Lucia Black Finch	EN
Mergus octosetaceus	Brazilian Merganser	CR
Mimus graysoni	Socorro Mockingbird	CR
Mimus trifasciatus	Floreana Mockingbird	EN
Myadestes palmeri	Puaiohi	CR
Nemosia rourei	Cherry-throated Tanager	CR
Neophema chrysogaster	Orange-bellied Parrot	CR

Nesoenas mayeri	Pink Pigeon	VU
Nesofregetta fuliginosa	Polynesian Storm-petrel	EN
Nesospiza wilkinsi	Wilkins's Bunting	EN
Ninox leventisi	Camiguin Boobook	EN
Ninox natalis	Christmas Boobook	VU
Ninox rumseyi	Cebu Boobook	EN
Ninox spilonotus	Romblon Boobook	EN
Nipponia nippon	Asian Crested Ibis	EN
Nisaetus bartelsi	Javan Hawk-eagle	EN
Nisaetus floris	Flores Hawk-eagle	CR
Nisaetus philippensis	North Philippine Hawk-eagle	EN
Nisaetus pinskeri	South Philippine Hawk-eagle	EN
Ognorhynchus icterotis	Yellow-eared Parrot	EN
Oreomystis bairdi	Akikiki	CR
Oriolus isabellae	Isabela Oriole	CR
Otus feae	Annobon Scops-owl	CR
Otus hartlaubi	Sao Tome Scops-owl	VU
Otus insularis	Seychelles Scops-owl	EN
Otus moheliensis	Moheli Scops-owl	EN
Otus thilohoffmanni	Serendib Scops-owl	EN
Oxypogon cyanolaemus	Blue-bearded Helmetcrest	CR
Oxypogon stuebelii	Buffy Helmetcrest	VU
Paradisaea decora	Goldie's Bird-of-paradise	VU
Passer hemileucus	Abd Al Kuri Sparrow	VU
Pauxi koepckeae	Sira Curassow	CR
Pedionomus torquatus	Plains-wanderer	CR
Penelope albipennis	White-winged Guan	EN
Penelope perspicax	Cauca Guan	EN
Penelopides mindorensis	Mindoro Hornbill	EN
Petroica dannefaerdi	Snares Tomtit	VU
Petroica multicolor	Norfolk Robin	EN
Petroica traversi	Black Robin	EN
Pezoporus occidentalis	Night Parrot	EN
Phalacrocorax featherstoni	Pitt Shag	EN
Phapitreron cinereiceps	Tawitawi Brown-dove	EN
Phibalura boliviana	Palkachupa Cotinga	EN
Philydor novaesi	Alagoas Foliage-gleaner	CR
Phylloscartes ceciliae	Alagoas Tyrannulet	CR
Phylloscopus amoenus	Sombre Leaf-warbler	VU
Pica asirensis	Asir Magpie	EN

Pipile pipile	Trinidad Piping-guan	CR
Pithecophaga jefferyi	Philippine Eagle	CR
Pitta anerythra	Black-faced Pitta	VU
Pitta superba	Superb Pitta	EN
Ploceus aureonucha	Golden-naped Weaver	EN
Ploceus batesi	Bates's Weaver	EN
Podiceps gallardoi	Hooded Grebe	CR
Podiceps taczanowskii	Junin Grebe	CR
Polyplectron katsumatae	Hainan Peacock-pheasant	EN
Pomarea dimidiata	Rarotonga Monarch	VU
Pomarea mendozae	Marquesan Monarch	EN
Pomarea whitneyi	Fatuhiva Monarch	CR
Poospiza rubecula	Rufous-breasted Warbling-finch	EN
Porphyrio hochstetteri	South Island Takahe	EN
Prioniturus verticalis	Sulu Racquet-tail	CR
Progne modesta	Galapagos Martin	EN
Prosobonia parvirostris	Tuamotu Sandpiper	EN
Pseudastur occidentalis	Grey-backed Hawk	EN
Pseudibis davisoni	White-shouldered Ibis	CR
Pseudobulweria aterrima	Mascarene Petrel	CR
Pseudobulweria becki	Beck's Petrel	CR
Pseudonestor xanthophrys	Maui Parrotbill	CR
Psittacula eques	Echo Parakeet	EN
Psophia obscura	Black-winged Trumpeter	CR
Pternistis ochropectus	Djibouti Francolin	CR
Pterodroma cahow	Bermuda Petrel	EN
Pterodroma deserta	Desertas Petrel	VU
Pterodroma madeira	Zino's Petrel	EN
Pterodroma magentae	Magenta Petrel	CR
Ptilinopus huttoni	Rapa Fruit-dove	CR
Ptilinopus rarotongensis	Rarotonga Fruit-dove	VU
Puffinus auricularis	Townsend's Shearwater	CR
Puffinus bryani	Bryan's Shearwater	CR
Puffinus heinrothi	Heinroth's Shearwater	VU
Puffinus myrtae	Rapa Shearwater	CR
Pyrrhura griseipectus	Grey-breasted Parakeet	EN
Pyrrhura orcesi	El Oro Parakeet	EN
Rallus wetmorei	Plain-flanked Rail	EN
Rhinoptilus bitorquatus	Jerdon's Courser	CR
Rhipidura malaitae	Malaita Fantail	VU

Rhynochetos jubatus	Kagu	EN
Rhyticeros narcondami	Narcondam Hornbill	EN
Rowettia goughensis	Gough Bunting	CR
Rukia ruki	Faichuk White-eye	EN
Sarothrura ayresi	White-winged Flufftail	CR
Sarothrura watersi	Slender-billed Flufftail	EN
Scepomycter winifredae	Winifred's Warbler	VU
Scytalopus canus	Paramillo Tapaculo	EN
Scytalopus iraiensis	Marsh Tapaculo	EN
Selasphorus ardens	Glow-throated Hummingbird	EN
Sitta ledanti	Algerian Nuthatch	EN
Spizaetus isidori	Black-and-chestnut Eagle	EN
Spizella wortheni	Worthen's Sparrow	EN
Sporophila iberaensis	Ibera Seedeater	EN
Strigops habroptila	Kakapo	CR
Symposiachrus boanensis	Black-chinned Monarch	CR
Synallaxis infuscata	Pinto's Spinetail	EN
Tanysiptera ellioti	Kofiau Paradise-kingfisher	VU
Taphrolesbia griseiventris	Grey-bellied Comet	EN
Terenura sicki	Orange-bellied Antwren	CR
Terpsiphone corvina	Seychelles Paradise-flycatcher	CR
Thalasseus bernsteini	Chinese Crested Tern	CR
Thapsinillas platenae	Sangihe Golden Bulbul	CR
Thaumatibis gigantea	Giant Ibis	CR
Thinornis novaeseelandiae	Shore Plover	EN
Thripophaga cherriei	Orinoco Softtail	VU
Thryophilus nicefori	Niceforo's Wren	CR
Todiramphus gambieri	Tuamotu Kingfisher	CR
Todiramphus godeffroyi	Marquesas Kingfisher	CR
Todiramphus ruficollaris	Mangaia Kingfisher	VU
Troglodytes monticola	Santa Marta Wren	CR
Troglodytes tanneri	Clarion Wren	VU
Turdus helleri	Taita Thrush	CR
Turdus xanthorhynchus	Principe Thrush	CR
Turnix hottentottus	Hottentot Buttonquail	EN
Turnix olivii	Buff-breasted Buttonquail	EN
Tyrannus cubensis	Giant Kingbird	EN
Tyto nigrobrunnea	Taliabu Masked-owl	VU
Vireo gracilirostris	Noronha Vireo	NT
Xenoglaux loweryi	Long-whiskered Owlet	EN

Zapornia olivieri	Sakalava Rail	EN
Zentrygon carrikeri	Tuxtla Quail-dove	EN
Zoothera turipavae	Guadalcanal Thrush	VU
Zosterops chloronothos	Mauritius Olive White-eye	CR
Zosterops ficedulinus	Sao Tome White-eye	EN
Zosterops luteirostris	Splendid White-eye	EN
Zosterops modestus	Seychelles White-eye	VU
Zosterops silvanus	Taita White-eye	EN
**REPTILES**		
Abronia campbelli	Campbell's Alligator Lizard	CR
Alligator sinensis	Chinese Alligator	CR
Batagur baska	Northern River Terrapin	CR
Batagur kachuga	Red-crowned Roofed Turtle	CR
Batagur trivittata	Burmese Roofed Turtle	CR
Bellatorias obiri	Arnhem Land Gorges Skink	CR
Borikenophis sanctaecrucis	Saint Croix Racer	CR
Celestus occiduus	Jamaica Giant Galliwasp	CR
Chelonoidis donfaustoi	Eastern Santa Cruz Giant Tortoise	CR
Chelonoidis duncanensis	Pinzón Giant Tortoise	VU
Chelonoidis guntheri	Sierra Negra Giant Tortoise	CR
Chelonoidis hoodensis	Española Giant Tortoise	CR
Chelonoidis microphyes	Volcán Darwin Giant Tortoise	EN
Chelonoidis phantasticus	Fernandina Giant Tortoise	CR
Chilabothrus argentum	Conception Bank Silver Boa	CR
Cnemaspis karsticola	Karst-dwelling Rock Gecko	VU
Cnemaspis psychedelica	Psychedelic Rock Gecko	EN
Conolophus marthae	Galápagos Pink Land Iguana	CR
Crocodylus intermedius	Orinoco Crocodile	CR
Crocodylus mindorensis	Philippines Crocodile	CR
Crocodylus siamensis	Siamese Crocodile	CR
Cuora yunnanensis	Yunnan Box Turtle	CR
Cyclura collei	Jamaican Iguana	CR
Cyclura lewisi	Grand Cayman Blue Iguana	EN
Cyrtodactylus chrysopylos	Cyrtodactylus chrysopylos	VU
Dierogekko inexpectatus		CR
Gavialis gangeticus	Gharial	CR
Hypsirhynchus ater	Jamaican Racer	CR
Hypsirhynchus melanichnus	La Vega Racer	CR
Liolaemus hellmichi	Hellmich's Tree Iguana	VU
Mabuya hispaniolae	Hispaniolan Two-lined Skink	CR
Mesoclemmys hogei	Hoge’s Side-necked Turtle	CR
Niveoscincus palfreymani	Pedra Branca Cool-skink	VU

Orraya occultus	Long-necked Northern Leaf-tailed Gecko	VU
Pholidoscelis corax	Censky's Ameiva	EN
Phrynocephalus golubewii	Phrynocephalus golubewii	CR
Psammobates geometricus	Geometric Tortoise	CR
Saltuarius eximius		EN
Spondylurus lineolatus	Hispaniolan Ten-lined Skink	CR
Spondylurus macleani	Carrot Rock Skink	CR
Tarentola boavistensis	Boavista Wall Gecko	VU
**AMPHIBIANS**		
Ameerega andina	La Planada Poison Frog	DD
Andinobates viridis	Green Poison Frog	CR
Arthroleptella rugosa	Rough Moss Frog	CR
Arthroleptides dutoiti	Du Toit's Torrent Frog	CR
Arthroleptis krokosua	Krokosua Squeaking Frog	NT
Atelopus angelito		CR
Atelopus ardila		CR
Atelopus chocoensis		CR
Atelopus ebenoides	Huila Stubfoot Toad	CR
Atelopus erythropus	Carabaya Stubfoot Toad	CR
Atelopus eusebiodiazi		CR
Atelopus exiguus		EN
Atelopus farci	Forest Stubfoot Toad	CR
Atelopus gigas		CR
Atelopus guanujo		CR
Atelopus halihelos	Morona-Santiago Stubfoot Toad	CR
Atelopus ignescens	Quito Stubfoot Toad	CR
Atelopus lynchi	Jambato de Lynch	CR
Atelopus mindoensis	Mindo Stubfoot Toad	CR
Atelopus minutulus	Colombian Stubfoot Toad	CR
Atelopus monohernandezii		CR
Atelopus nicefori	Niceforo's Stubfoot Toad	CR
Atelopus onorei		CR
Atelopus orcesi		CR
Atelopus pachydermus	Schmidt's Stubfoot Toad	CR
Atelopus pedimarmoratus	San Isidro Stubfoot Toad	CR
Atelopus peruensis	Peru Stubfoot Toad	CR
Atelopus petriruizi	Peters' stubfoot toad	CR
Atelopus pictiventris	Painted Stubfoot Toad	CR
Atelopus planispina	Flat-spined Atelopus	CR
Atelopus podocarpus		CR
Atelopus quimbaya		CR

Atelopus sernai		CR
Atelopus simulatus		CR
Atelopus sonsonensis		CR
Atelopus subornatus	Bogota Stubfoot Toad	CR
Dryophytes suweonensis	Suweon Treefrog	EN
Duellmanohyla uranochroa	Costa Rica Brook Frog	EN
Gastrotheca angustifrons	Pacific Marsupial Frog	VU
Gastrotheca antomia		CR
Gastrotheca dendronastes	Rio Calima Marsupial Frog	VU
Hyloxalus fascianigrus	Rana Saltarina de Brazalete	VU
Leiopelma hamiltoni	Stephens Island Frog	VU
Leptodactylus fallax	Mountain Chicken	CR
Leptophryne cruentata	Bleeding Toad	CR
Lithobates vibicarius	Rancho Redondo Frog	VU
Nannophryne cophotis	Paramo Toad	CR
Philautus jacobsoni	Jacobson's Bubble-nest Frog	CR
Pristimantis albericoi	Pristimantis albericoi	CR
Pristimantis bernali	Argelia Robber Frog	CR
Pristimantis cacao	Cacao Robber Frog	EN
Pristimantis chrysops		EN
Pristimantis deinops		EN
Pristimantis diaphonus	Rio Calima Robber Frog	CR
Pristimantis diogenes		VU
Pristimantis molybrignus	Uribe Robber Frog	CR
Pristimantis phragmipleuron	Sugar Robber Frog	CR
Pristimantis xylochobates		CR
Raorchestes chotta	Small Bushfrog	DD
Raorchestes resplendens	Resplendent Shrubfrog	CR
Rhaebo colomai	Carchi Andes Toad	EN
Rhinoderma rufum	Northern Darwin's frog	CR
Strabomantis necerus	Mindo Robber Frog	VU
Telmatobius fronteriensis		DD
Telmatobius pefauri	Arico Water Frog	CR
